# Myxoid Plexiform Fibrohistiocytic Tumor Masquerading as Ganglion Cyst: A Case Report and Literature Review

**DOI:** 10.1155/2017/5370894

**Published:** 2017-01-31

**Authors:** Chih-Yi Liu, Jui Lan, Hsuan-Ying Huang

**Affiliations:** ^1^Division of Pathology, Sijhih Cathay General Hospital, New Taipei City, Taiwan; ^2^College of Medicine, Fu Jen Catholic University, New Taipei City, Taiwan; ^3^Departments of Pathology, Chang Gung Memorial Hospital and Chang Gung University College of Medicine, Kaohsiung, Taiwan

## Abstract

*Background*. Plexiform fibrohistiocytic tumor is a distinctive mesenchymal neoplasm of low-grade malignancy, with the capacity for biphasic differentiation toward a fibroblastic or histiocyte-like morphology. Clinically, these lesions affect different areas of the body and appear as painless, slowly growing, dermal or subcutaneous masses. To date, only three cases of myxoid variant have been reported in the English literature.* Case Presentation*. A 45-year-old female patient presented with a subcutaneous nodule in the right popliteal fossa. The initial impression was a benign ganglion cyst. The soft tissue tumor has been treated by local excision. The histopathological and the immunohistochemical findings supported the diagnosis of myxoid plexiform fibrohistiocytic tumor. The postoperative course was uneventful, and the patient received regular follow-up examination. She is alive without any recurrence.* Conclusions*. This case demonstrates how to distinguish this distinctive plexiform fibrohistiocytic tumor from other problematic soft tissue tumors. It is also remarkable for its rarely reported extensive myxoid change. Currently, there is no clear-cut correlation between the clinicopathologic findings and the behavior of this unusual variant.

## 1. Introduction

Plexiform fibrohistiocytic tumor (PFHT) comprises a peculiar group of lesions displaying multinodular or plexiform proliferation of histiocyte- and fibroblast-like cells in variable proportions, with or without multinucleated giant cells [1, 2]. It has a predilection for children and young adults but can occur at any age. Plexiform fibrohistiocytic tumor typically presents as a slowly growing, painless mass. The tumors usually are centered in the superficial soft tissues. Cure after complete surgical excision seems to be the typical clinical course. However, local recurrence and occasional metastatic behavior have been documented [2, 3]. Histologically, only three cases of myxoid plexiform fibrohistiocytic tumor have been reported in the literature [4–6]. We add a case of myxoid plexiform fibrohistiocytic tumor arising in the popliteal fossa. The clinical impression before treatment is subcutaneous ganglion cyst. Given the unusual histological features, myxoid plexiform fibrohistiocytic tumor should also be considered in the differential diagnosis of a soft tissue tumor with myxoid matrix.

## 2. Case Presentation

**Figure 1:**
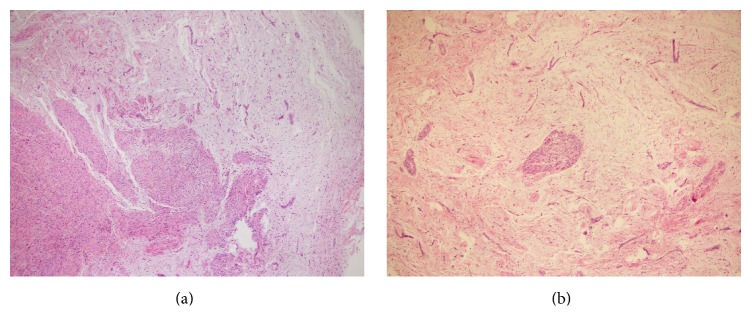
Histopathological findings. Plexiform proliferation of tumor cells with micronodules set in a myxoid background (hematoxylin and eosin stain; ×40 magnification).

**Figure 2:**
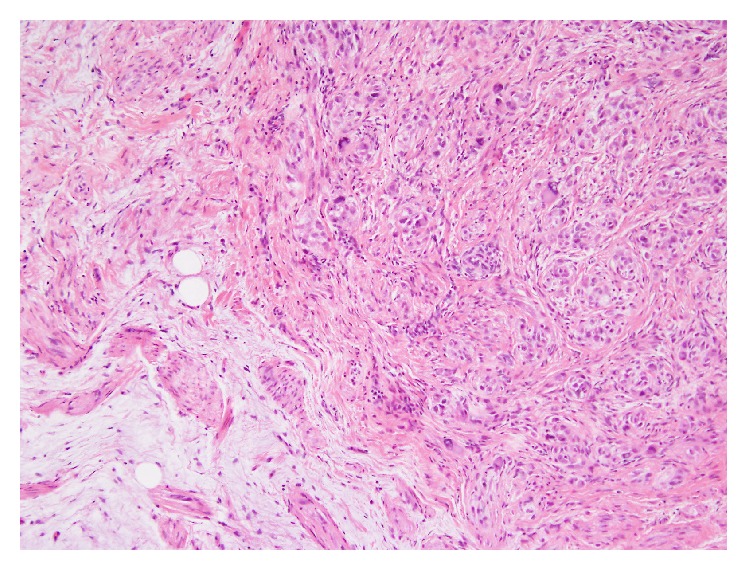
Histopathological findings. Cellular areas composed of histiocyte-like cells and spindled fibroblast-like cells at the periphery (hematoxylin and eosin stain; ×200 magnification).

A 45-year-old female patient presented with a painless and nodular lesion in the right popliteal fossa. The tumor grew slowly in the past three months. In April 2015, she visited the plastic surgery clinic of Sijhih Cathay General Hospital in New Taipei City. The preoperative impression was ganglion cyst of the popliteal fossa. The surgeon performed local excision of the tumor. Histopathologically, it showed a myxoid nodule with ill-defined borders, up to 20 mm in maximum dimension. The tumor exhibited plexiform and nodular growth of both myofibroblast-like spindle cells and epithelioid cells, set in a prominent myxoid stroma (Figures 1(a) and 1(b)). The cellular areas were composed of epithelioid and histiocyte-like cells with few multinucleated giant cells (Figure 2). Evident cytologic atypia was not seen. There was a very low mitotic activity with less than one mitotic figure per 10 high power microscopic fields. Tumor necrosis and vascular space permeation were not identified.

**Figure 3:**
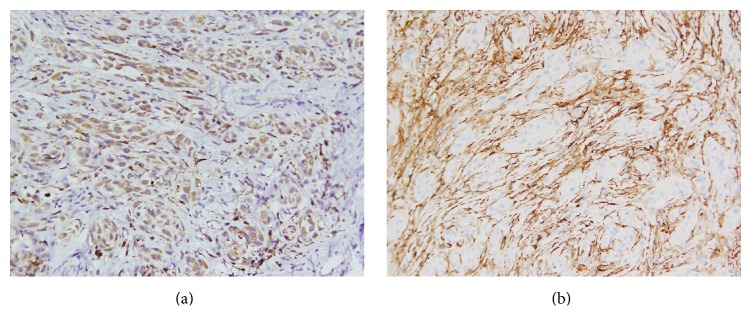
Immunohistochemical study. Immunohistochemical staining for (a) CD68, diffusely positive in epithelioid or histiocyte-like cells, and (b) CD34, diffusely positive in spindle or fibroblast-like cells (×200 magnification).

Immunohistochemically, the epithelioid cells were positive for CD68, while the spindle cells were diffusely positive for CD34 and focally positive for smooth muscle actin (SMA) (Figures 3(a) and 3(b)). Notable immunoreactivity was not seen for S-100 protein, GFAP, Bcl-2, cytokeratin (AE1/AE3), or EMA. The above immunophenotype was consistent with the diagnosis of myxoid plexiform fibrohistiocytic tumor. The tumor was excised with free margins. The patient did not accept further treatment. After the surgery, she has received follow-up examination twice in one year. She is alive without any local recurrence or distant metastasis.

## 3. Discussion

Initially presented in a case series of 65 patients, Enzinger and Zhang described a morphologically and immunohistochemically distinct subset of fibrohistiocytic neoplasm that occurred chiefly in children and young adults [1]. Histologically, these superficial soft tissue tumors were characterized by multinodular or plexiform proliferation of histiocyte- and fibroblast-like cells associated with multinuclear giant cells. Enzinger and Zhang defined the diagnostic entity of plexiform fibrohistiocytic tumor. Over the past two decades, the clinicopathologic characteristics of PFHT have been further analyzed in four large case series [1–3, 7]. These case series reveal that PFHT has a predilection for children and young adults but can occur at any age [8]. Besides, the possibly histogenetic link among PFHT and cellular neurothekeoma has also been documented [9].

Clinically, PFHT usually presents as a painless, slowly growing soft tissue mass that is situated in the dermis and subcutis [8]. There is a wide anatomical distribution. The most common site is the upper limb, especially forearm, elbow, and shoulder, followed by lower extremities, trunk, head, and neck [10, 11]. Although PFHT may develop at any age, the tumor occurs most frequently in children and adolescents, with about 70% of patients being below 20 years old [10]. A minority of cases have been found to be of congenital origin [7]. Most PFHTs measure less than 3 cm in the greatest diameter. The smallest lesion recorded was 0.3 cm and the largest was 8.5 cm [8]. These lesions may affect the subcutaneous tissue and occasionally extend into the skeletal muscle. Limits are vaguely defined, with irregular or slightly lobulated borders [11].

Histopathologically, PFHTs are typically composed of two main components, histiocyte- and spindled fibroblast-like cells in variable proportions. These tumors have been morphologically divided into three groups: fibroblastic, histiocytic (often with osteoclast-like giant cells), and mixed type [3, 7]. The amount of each cell type present in a tumor varies in cases, and the subtype is defined according to the predominant cell type. PFHTs are characterized by a plexiform and infiltrative growth pattern as well [7]. Osteoclast-like giant cells are often observed in PFHT, particularly in the histiocytic subtype. However, PTHF does not always contain giant cells, as originally observed [7, 11]. Immunohistochemically, the histiocytic components and the osteoclast-like giant cells exhibit CD68 positivity. The fibroblast-like cells are usually positive for SMA, but negative for CD68. PFHT is negative for cytokeratin, desmin, HMB45, and S-100 protein. Reactivity for CD34 may be occasionally observed in the spindle cells.

Similar to the previous case series, our case showed a subcutaneous tumor formed by biphasic proliferation of fibroblast-like and histiocyte-like cells. It is also remarkable for the unusual features of extensive myxoid change. To date, only three myxoid PFHTs have been reported [4–6]. These peculiar myxoid PFHTs arose in a 58-year-old man, a 24-year-old woman, and a 58-year-old man, respectively. Some authors suggested that myxoid changes might reflect a distinctive pattern of PFHTs in older individuals [4]. On the other hand, myxoid changes in young adults might represent degeneration, likely related to the longstanding process commonly seen in PFHTs [5]. The differential diagnosis of myxoid PFHTs depends on the predominant histological pattern and includes especially those tumors with a prominent plexiform pattern. Cellular neurothekeoma seems to be a lesion related to PFHT, particularly when PFHT is deep dermal or superficial subcutaneous [7]. Cellular neurothekeoma and PFHT are both made up of S-100-negative cells, which are sometimes positive for CD68 [9]. However, cellular neurothekeoma is rarely myxoid and lacks the osteoclast-like giant cells of PFHT [4]. The plexiform growth pattern and the myxoid matrix may raise a concern for neurogenic tumor such as plexiform neurofibroma. Plexiform neurofibroma typically exhibits tortuous masses of expanded nerve branches in a fibromyxoid background, with the presence of S-100 protein positivity [10]. Owing to the bland morphology and the abundant myxoid matrix, low-grade fibromyxoid sarcoma has to be considered. Low-grade fibromyxoid sarcoma usually shows whorled distributions of fibroblasts with rosette formation and pronounced fibrous zones. Recognition of the characteristic cellular components and application of appropriate immunohistochemical panels usually lead to a correct diagnosis.

PFHT is now considered as a superficial soft tissue tumor with a low to intermediate malignant potential [8]. Although the biological potential of PFHT is not possible to predict, the prognosis is generally favorable after complete surgical excision [10]. However, local recurrence rate of up to 40% has been reported [2]. Six percent of cases have metastasized to regional lymph nodes, and 2–19% have metastasized to lungs [12]. Unfortunately, no reliable histological parameters could predict the risk of local recurrence, regional lymph node, or systemic metastases [10]. Long-term follow-up is recommended for these patients.

## 4. Conclusions

In summary, the current study presents a rare case of PFHT with extensive myxoid change. The clinical presentation masquerades as benign ganglion cyst of the popliteal fossa. The distinct histopathological pattern must be included in the differential diagnosis of myxoid soft tissue tumors, while the impact on tumor behavior remains unclear. Complete surgical excision and careful clinical follow-up are necessary because of the possibility of local recurrence and occasional distant metastasis.
